# Emerging Roles of Wnt Ligands in Human Colorectal Cancer

**DOI:** 10.3389/fonc.2020.01341

**Published:** 2020-08-14

**Authors:** Xiaobo Nie, Huiyang Liu, Lei Liu, Yan-Dong Wang, Wei-Dong Chen

**Affiliations:** ^1^Key Laboratory of Receptors-Mediated Gene Regulation and Drug Discovery, People's Hospital of Hebi, School of Medicine, Henan University, Henan, China; ^2^State Key Laboratory of Chemical Resource Engineering, College of Life Science and Technology, Beijing University of Chemical Technology, Beijing, China; ^3^Key Laboratory of Molecular Pathology, School of Basic Medical Science, Inner Mongolia Medical University, Hohhot, China

**Keywords:** colorectal cancer, Wnts, canonical Wnt signaling pathway, non-canonical Wnt signaling pathway, Wnt-based therapeutics

## Abstract

Colorectal cancer (CRC) is the fourth leading cause of cancer death worldwide, and constitutive activation of the Wnt signaling pathway is universal in most CRC cases. Wnt ligands (Wnts) are secreted glycoproteins and fundamentally essential for the transduction of Wnt signaling pathway. However, the 19 members of Wnts in humans imply a daunting complexity of Wnt signaling and biological effects, and our understanding of their roles in CRC tumorigenesis is still quite rudimentary. This review will give an overview of the structural characteristics and maturation process of Wnts. The expression pattern of all human Wnts in CRC tissues, including Wnt1, Wnt2, Wnt2b, Wnt3, Wnt3a, Wnt4, Wnt5a, Wnt5b, Wnt6, Wnt7a, Wnt7b, Wnt8a, Wnt8b, Wnt9a, Wnt9b, Wnt10a, Wnt10b, Wnt11, and Wnt16, and their relationship with the tumorigenesis and the progression of CRC will be specifically summarized separately. Despite certain challenges, Wnt-based therapeutics for CRC emerge continuously and some are now in clinical trials. In conclusion, a deep understanding of Wnts is very helpful for a better management of this disease.

## Introduction

Colorectal cancer (CRC) is the third most common malignancy and the fourth leading cause of cancer-related mortality worldwide, with more than 1.4 million new cases and 800,000 cancer-related deaths annually. The occurrence of CRC can be attributed to multiple lifestyle risk factors, such as diets high in fat and cholesterol, lack of exercise, excessive alcohol consumption and smoking, and other uncontrollable risk factors, including aging, type 2 diabetes, personal history of colonic polyps or inflammatory bowel disease, and some CRC-related hereditary syndromes. The routine use of fecal occult blood test, colonoscopy, and image evaluation has significantly improved the detection of CRC, and improved treatment options such as targeted therapy and immunotherapy have raised the 5-year survival rate to 65% for patients with CRC (1). Unfortunately, the tumor often reaches to the advanced stage or metastasizes without noticeable symptoms, and about 25% of CRC patients have metastatic diseases at initial diagnosis and their prognosis is still very poor (2). Therefore, an improved understanding of the underlying molecular mechanisms will contribute to the diagnostic and the therapeutic management of CRC.

## Roles of Wnt Signaling in Tumorigenesis and the Progression of CRC

CRC is a highly heterogeneous disease, which is attributed to the complex interactions between genetic predisposition and environmental factors, and abnormalities in several crucial signal transduction pathways, such as Notch, TGFβ-Smads, Hedgehog, JAK-STAT, Ras-MAPK, PI3K-Akt, Wnt, p53, and DNA mismatch repair signaling pathways, play important roles in the initiation and the progression of CRC (3). Among them, Wnt signaling pathway attracts more attention due to its crucial role in a variety of biological processes, such as embryogenesis and tissue homeostasis. Abundant studies have proved that excessive activation of Wnt signaling was a major culprit in the carcinogenesis of most human malignancies, including CRC (4, 5). A genome-scale analysis has identified that more than 90% of CRC patients carried mutations of one or more downstream components of the Wnt signaling pathway, especially the loss-of-function mutations of adenomatous polyposis coli (APC) and the activating mutations of β-catenin or the extreme overexpression of some members such as frizzled (Fzd) receptors (6). Moreover, mutations of Wnt-dependent components, such as activating mutations of R-spondin (RSPO) family members and secreted Wnt agonists, occur in 10% of CRC cases carrying the wild-type APC allele (7). Additionally, the loss-of-function mutations of E3 ubiquitin ligases ring-finger protein 43 (RNF43), which lead to the excessive activation of Wnt signaling by blocking the ubiquitin-mediated degradation of Fzd receptors and LRP5/6 coreceptors, are dependent on Wnt secretion and frequently detected in CRC cases (8).

**Figure 1:**
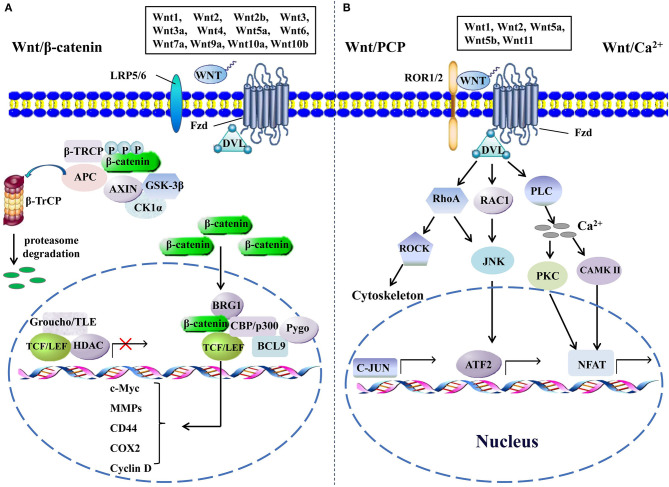
The canonical and the non-canonical Wnt signaling pathways. **(A)** In canonical Wnt signaling, β-catenin is phosphorylated by GSK-3β and CK1α in the absence of Wnts, followed by ubiquitination by β-TrCP and targeting for proteasomal degradation, without nuclear β-catenin; a repressive complex containing TCF/LEF and Groucho/TLE subsequently recruits HDACs to inhibit the transcriptional activation of β-catenin target genes. Conversely, the activation of canonical Wnt signaling is initiated from the binding of Wnts to Fzd and co-receptor LDL receptor-related protein (LRP5 or LRP6); then, the DVL is phosphorylated by GSK-3β and CK1α and begins to form a polymer that can inactivate the destruction complex through recruiting AXIN and GSK3β. Thereby, the accumulated β-catenin in the cytoplasm localizes to the nucleus and forms complexes with co-regulators of transcription factors such as TCF/LEF by removing Groucho/TLE complexes and recruiting transcriptional co-activators, including CBP/p300, BRG1, BCL9, and Pygopus. Next, downstream genes including cyclin D, MMPs, c-Myc, COX2, CD44, etc., are activated and give rise to the changes of the series of cellular activities, such as excessive cell proliferation, motility, and polarity. **(B)** The β-catenin-independent non-canonical Wnt signaling is initiated by binding certain Wnts and could regulate cellular polarity and migration-related signaling pathways. In the Wnt/PCP pathway, Wnts bind to the ROR1/2-Fzd complex to activate DVL, DVL binds to small Rho GTPases such as RAC1 and RhoA, RhoA and RAC1 together trigger JNK, and RhoA activates ROCK alone. This leads to the asymmetric cytoskeletal organization and/or coordination of cellular polarization *via* activating the transcription factors, such as c-JUN and ATF2. The Wnt/Ca^2+^ signaling triggers PLC activity and subsequently induces calcium influx; then, elevated Ca^2+^ activates several calcium-dependent signaling pathways, such as PKC and Ca^2+^/CAMKII, which finally leads to the accumulation of transcription factor NFAT in the nucleus. ATF2, activating transcription factor 2; BCL9, B-cell CLL/lymphoma 9; BRG1, brahma-related gene 1; β-TRCP, β-transducin repeat-containing protein; CAMKII, calmodulin-dependent protein kinase II; CK1α, casein kinase 1; COX2, cytochrome c oxidase subunit 2; DVL, disheveled; GSK-3β, glycogen synthase kinase; HDACs, histone deacetylases; JNK, JUN N-terminal kinase; LEF, lymphoid enhancer-binding factor; MMPs, matrix metalloproteinases; NFAT, nuclear factor of activated T cells; PCP, planar cell polarity; PKC, protein kinase C; PLC, phospholipase C; ROCK, rho kinase; ROR1/2, receptor tyrosine kinase-like orphan receptor 1/2; TCF, T-cell factor; TLE, transducin-like enhancer protein.

The activation of the Wnt signaling pathway, depending on the alteration of the Wnt pathway components and their functions, is indispensable for the initiation, the progression, and the metastasis of CRC. The Wnt signaling pathway transduction may be interrupted or exceedingly activated when the expression levels of crucial components change, especially in tumorigenesis (9). Even though the majority of the Wnt signaling pathway components have been determined, their functions in a specific tumor type or microenvironment remain intriguingly complicated and need to be understood deeply. The Wnt signaling pathway is mainly divided into β-catenin-dependent canonical signaling pathway, independent non-canonical Wnt/planar cell polarity, and Wnt/Ca^2+^ signaling pathways (Figure 1). It is still unclear by which mechanism Wnts choose to activate one specific signaling. A reasonable explanation is that the cell type and the signaling components expressed in cells may dictate the specificity of the signaling cascade and the downstream effectors (10). The identified transduction processes of the canonical Wnt signaling mainly include the secretion of Wnts, identification of Wnt coreceptors, silencing of β-catenin destruction complex, translocation of β-catenin into nucleus, recruitment of co-factors, and activation of target genes. An aberrant regulation of any of the steps mentioned above in canonical Wnt signaling could contribute to the development of human malignancies, and several studies have well-documented the impact of Wnt signaling on the carcinogenesis of CRC (9, 11). Besides that, abnormal feedback regulation of the Wnt pathway is also involved in the carcinogenesis of CRC. For instance, AXIN2 and DKK1 are direct targets and feedback inhibitors of the Wnt pathway in normal cells, whereas their inhibitory effect on activated Wnt signaling in CRC cells is invalid, and instead they become the promoters of CRC metastasis by activating epithelial–mesenchymal transition (EMT) pathways (12). Recently, Kang and colleagues found that phopholipase D isozymes, the direct targets and positive feedback regulators of the canonical Wnt pathway, could promote Wnt-driven growth and invasion of CRC cells (13). Moreover, complex interactions of Wnt pathways and many other signaling pathways, such as NF-κB, RAS-extra-cellular signal regulated kinase, and hypoxia-inducible factor-1α pathways, are commonly observed in most tissue types, and the aberrations within these pathways also contribute to the development of CRC, increasing the difficulty of designing better interventions against it (14–16). Overall, dysregulation of Wnt signaling is an important pathogenetic basis of CRC, and revealing detailed mechanisms of its action is critical for the treatment of this disease.

As key factors to initiate the activation of Wnt signaling pathway, Wnts are expressed in all metazoan species, and humans carry 19 independent members sharing 40–90% amino acid sequence identity with each other. All Wnts are secreted glycosylated lipid-modified molecules. However, little is known regarding what determines the generation of various Wnts and the subsequent Wnt signaling pathways. Although the aberrant expression of Wnts is not the culprit for triggering majority of CRC, elucidating their specific roles in colorectal carcinogenesis is still beneficial for the diagnosis and the prevention of CRC. In the following parts, we will discuss the current insights into the characteristics of human Wnts and elaborate their roles in the pathogenesis of CRC. In addition, we will summarize the latest progress on the treatment of CRC by targeting Wnts.

## The Structural Characteristics of Wnts

The structure of Wnts has been elaborately summarized previously (17, 18). In brief, Wnts consist of 350–400 amino acid residues and are ~40 kDa in size. The amino-terminal signal sequences are mainly hydrophobic amino acids in different lengths for secretion and may be cleaved for maturation (19). The amino terminus is predicted to determine which Wnt signaling will be activated (20), whereas other hypothesis suggests that Wnt signaling activity is perhaps conferred by specific cellular context based on the observations that some non-canonical ligands, such as Wnt5a and Wnt11, can activate the canonical Wnt/β-catenin in a certain context (19). Wnts also contain about 22 conserved cysteine residues which are postulated to form intramolecular S–S bonding and maintain the secondary structure. The high-resolution structural information of Wnts has puzzled scientists for decades, and this problem was solved by Garcia and colleagues. They crystallized the *Xenopus* Wnt8 (*X*Wnt8) with the cysteine-rich domain (CRD) of Fzd8 and revealed that the amino-terminal domain (NTD) was composed of six α-helices from residues 1–250 and two β-strand hairpins, with five pairs of conserved cysteine residues to form disulfide bonds, and the carboxyl-terminal domain (CTD) from residues 261–338 rich in cysteine residues and constructed from four α-helices and two β-strand hairpins stabilized by six disulfide bonds. A serine residue (Ser187 of *X*Wnt8) attached to a lipid group in NTD binds to a deep groove in Fzd8-CRD, and index finger forms conserved hydrophobic residues in CTD that contacts with a depressed region of Fzd8-CRD (21). Furthermore, a recent structural analysis on human Fzd5 and Fzd7 CRD also uncovered that the unsaturated fatty acyl group in Wnts was the common molecular mechanism for the recognition of multiple Fzd receptors and the subsequent dimerization (22). However, no structural information for human Wnts has been analyzed because the complicated lipid modification and combination with carrier proteins restrict their purification in natural form (23, 24).

## The maturation of Wnts

Upon translation, Wnts are targeted to the endoplasmic reticulum where glycosylation and acylation take place. The number of glycosylation attachment endows the diversity of Wnts and controls over the subsequent acylation, folding, and secretion. Acylation is essential for the activity of Wnts, and nearly all Wnts are modified with unsaturated fatty acid such as palmitic acid at a conserved serine residue by acyltransferase called porcupine (*PORCN*) (67). Deletion or mutation in any porcupine isoforms will block the whole Wnt signaling transduction and lead to embryonic lethality in mice (68), and mutations in X-linked *PORCN* could cause a developmental disorder named focal dermal hypoplasia in human (69, 70). Additionally, other modifications such as O-sulfation at specific tyrosine residues are necessary for the hetero-oligomer of certain Wnts and canonical Wnt signaling activity (71). In Golgi apparatus, a conserved transmembrane protein Wntless (Wls) binds to Wnts and accompanies them to the cell surface for secretion (72, 73), and a conserved serine residue (in Wg S239) in Wnts is essential for their recognition by Wls (74). Coombs et al. proposed that vacuolar acidification was also required to release Wnts from Wls in secretory vesicles (75), and this anterograde secretory process also relies on p24 proteins which function as conserved cargo receptors (76). The release of Wnts from cells also depends on a lipocalin family member of extracellular transport proteins, which binds to Wnts with high affinity and maintains their solubility and activity (23). Once reaching the surface of receptor cells by autocrine or paracrine fashion, Wnts encounter multiple interacting molecules such as polyanionic compounds, glycans, and a myriad of protein-binding partners including Wnt inhibitory factor (WIF) and Fzd receptors to initiate the Wnt pathways, whereas massive questions concerning the switch mechanism of Wnt pathways needed to be answered.

## Roles of Wnts in Tumorigenesis and the Progression of CRC

Although the function of Wnts varies in the initiation and the progression of CRC, their expression pattern can serve as an important diagnostic or prognostic indicator for patients with CRC. In the following paragraphs, the biological functions of each human Wnt in colorectal carcinogenesis will be discussed separately (Table 1).

**Table 1:** Oncogenic and tumor suppressor Wnts regulating the canonical and the non-canonical Wnt signaling pathways in the pathogenesis of colorectal cancer (CRC).

**Wnts**	**Expression level**	**Effect**	**Type of Wnt signaling**
Wnt1	Decreased (25)	Oncogene	Canonical (26)
	Increased (27)		Non-canonical (28)
Wnt2	Increased (29–31)	Oncogene	Canonical (32)
			Non-canonical (33)
Wnt2b	Increased (34)	Oncogene	Canonical (34)
Wnt3	Increased (35)	Oncogene	Canonical (35)
Wnt3a	Increased (36)	Oncogene	Canonical (37)
		Tumor suppressor	Canonical (38)
Wnt4	Increased (39)	Oncogene	Canonical (39, 40)
Wnt5a	Decreased (41, 42)	Tumor suppressor	Canonical (43)
			Non-canonical (44)
	Increased (45, 46)	Oncogene	Canonical (47)
			Non-canonical (48)
Wnt5b	Increased (49, 50)	Oncogene	Non-canonical (50)
Wnt6	Increased (51, 52)	Oncogene	Canonical (53, 54)
Wnt7a	Increased (49, 55)	Oncogene	No data in CRC
		Tumor suppressor	Canonical (56)
Wnt7b	No data in CRC		
Wnt8a	No data in CRC		
Wnt8b	No data in CRC		
Wnt9a	Decreased (57)	Tumor suppressor	Canonical (58)
Wnt9b	No data in CRC	Oncogene (59)	No data in CRC
Wnt10a	Increased (60, 61)	Oncogene	Canonical (60, 61)
	Decreased (62)		No data in CRC
Wnt10b	No data in CRC	Oncogene	Canonical (63)
Wnt11	Increased (64)	Oncogene	Non-canonical (65)
Wnt16	No data in CRC	Oncogene (66)	No data in CRC

Wnt1 is one of the ligands which mainly activate the canonical Wnt signaling cascade. The transcriptional activation of the *Wnt1* (int-1) gene was firstly proved to be the initiating step in mammary gland hyperplasia and adenocarcinomas in mice (77). The transient or stable expression of Wnt1 could induce the formation of β-catenin–LEF1 complex and the persistent activation of canonical Wnt signaling in CRC cells (78). CRC cells expressing Wnt1 are resistant to cancer chemotherapy, and Wnt1 could inhibit the apoptosis by activating β-catenin/TCF transcription (26). Intriguingly, a latest study reported that exosomal Wnt1 could largely enhance the proliferation and the migration of CRC cells through activating the non-canonical Wnt signaling (28). Blockade of Wnt1 by WIF-1 or its antibody induced a significant apoptosis of human CRC cells containing mutations of APC, CTNNB1, and AXIN2 (79). Moreover, the ectopic expression of microRNA (miR)-200b-3p and miR-185 could significantly inhibit the proliferation and induce the apoptosis of CRC cells by targeting the canonical Wnt1/β-catenin signaling (80, 81). However, researches on the Wnt1 expression in human CRC tissues have yielded some conflicting results that the decrease or the increase of Wnt1 expression was detected in CRC tissues compared with normal colorectal mucosa (25, 27), and a study even found that the expression level of Wnt1 was decreased in human CRC tissues and CRC mice, whereas Wnt1 knockdown could still dramatically decrease the cell migration and the invasion of human CRC cells, and β-catenin expression was also enhanced in the tumors, indicating that Wnt1 expression could be regulated by more complicated mechanisms during CRC tumorigenesis (82).

Wnt2 is also an oncogene with a potential to activate the canonical Wnt signaling during CRC tumorigenesis (83). Cancer-associated fibroblasts were identified as the main source of Wnt2, and Wnt2 could enhance the tumor growth and the invasion of CRC in a paracrine fashion (32). Meanwhile, the invasive activity of CRC cells was also induced by Wnt2 through a non-canonical Wnt pathway coupled to GSK-3β and c-Jun/AP-1 signaling (33). Wnt2 was expressed at high levels in all CRC tissue samples at different stages, including premalignant colorectal polyps and liver metastasis, and high Wnt2 expression levels indicated poor prognosis in human CRC, although this upregulation was not due to the mutation in its coding region (29–32). Another analysis demonstrated that Wnt2 was upregulated in the progression from colorectal adenoma to carcinoma, and *in situ* hybridization showed that Wnt2 was expressed predominantly in macrophages in the lamina propria/stroma regions (84). Depletion of endogenous Wnt2 or neutralizing secreted Wnt2 could suppress the proliferation of CRC cells by targeting the canonical Wnt signaling. Galectin-3 (Gal-3) is a multifunctional carbohydrate-binding protein and proven to interact with β-catenin (83). The combined inhibition of Wnt2 and Gal-3 has synergistic effects on destabilizing β-catenin and induce the apoptosis of human CRC cells (85). Wnt2 and Fzd7 are key players in CRC progression. In a recent study, Kalhor and colleagues assessed the three-dimensional structure of human Wnt2-Fzd7 CRD complex *via* bioinformatics approaches, and the data demonstrated a unique dynamic behavior of Wnt2 upon binding to Fzd7, which is highly useful in targeted therapy for Wnt2-related cancers (86). Wnt2b is a paralogue of Wnt2, with amino acid identity of 70%, and Wnt2b shows a different expression pattern in human malignancies (87). However, the role of Wnt2b in CRC development has been rarely reported; only a recent study demonstrated that Wnt2b was significantly increased in colon cancer cells compared with normal colon epithelial cells, and inhibiting the activity of a CRC-promoting nuclear factor, estrogen receptor, could significantly decrease the Wnt2b/β-catenin signaling in colon cells (34).

Wnt3 and Wnt3a are highly homologous proteins with 85% amino acid sequence identity. However, 15% of difference exerts a great influence on protein structure and dynamics under the same condition, which eventually leads to different biological functions (88). Wnt3 was highly expressed in colon cancer tissues, and autocrine Wnt3 secretion *via* Evi/Wls was required to maintain the Wnt activity in colon cancer cells. Interfering with the secretion of Wnt3 could impair the growth of colon cancer cells *in vitro* and *in vivo* (35). We previously reported that gastric tumors also expressed elevated levels of Wnt3, and silencing Wnt3 in gastric cancer cells could block cell proliferation and induce apoptosis through targeting the canonical Wnt pathway (89). Recently, we found that the upregulation of Wnt3 in human CRC cell lines was essential for CRC progression. The knockdown of Wnt3 in CRC cells suppressed the proliferation but enhanced the sensitivity to chemotherapeutics by inhibiting the canonical Wnt pathway and glycolytic pathway (90). A83-01 is a selective inhibitor of TGF-β receptor; it was shown to inhibit EMT in HER2-overexpressing breast cancer cells by interfering the TGF-β-induced upregulation of Wnt3 (91), whereas its application in treating CRC has not been reported.

The expression of Wnt3a was also elevated in CRC tissues and associated with EMT, for advanced stages as well as poor prognosis (36). Moreover, the expression of Wnt3a was higher in the primary sites than that in the metastatic sites of CRC tissues, suggesting that the expression of Wnt3a was induced in the initial period of CRC rather than emerging as the cancer progressed. The expression level of Wnt3a in primary tumors was positively correlated with lymph node involvement and the expression of certain metastatic related genes (92). Consistently, Schinzari et al. demonstrated that the concentration of secreted Wnt3a was much higher in conditioned medium from normal or tumor tissues obtained from CRC patients than that from healthy donors (93). Therefore, approaches to inhibit Wnt3a expression have been proposed to suppress CRC, and metformin was proved to attenuate the cell stemness and EMT in CRC cells by inhibiting the Wnt3a/β-catenin pathway (37). However, some exceptions revealed that the role of Wnt3a in CRC was not coupled. In a more recent study, Wnt3a was found to inhibit the proliferation and the migration capacities of human colon myofibroblasts, the latter of which has been recognized to promote CRC progression (38). Thus, the variable role of Wnt3a is probably due to the specific molecular and cellular characteristics of different CRC subgroups and its context-dependent nature.

Wnt4 regulates many crucial embryonic and developmental pathways through activating the canonical Wnt signaling and non-canonical mechanisms (94, 95). Dysregulation or variants of *Wnt4* gene may disturb these host networks, leading to the malignant transformation of cells and the occurrence of many cancers. For example, Al-Tassan et al. have identified novel risk variants for CRC near *Wnt4* gene (96). Interestingly, exosomes derived from hypoxic CRC cells could transfer Wnt4 to normoxic CRC cells to enhance pro-metastatic behaviors and promote angiogenesis in endothelial cells by activating the canonical Wnt signaling (39, 40), which demonstrates a novel mechanism for the development of CRC. Excitingly, the structural dynamic behavior of Wnt4 protein was analyzed by a comparative computational study, and a foundation for designing new Wnt4 inhibitors to combat its irregularities was established (97). Recently, tetramethylthiuram disulfide, an important pesticide extensively used in agriculture, was proved to reduce the growth performance of chickens by inhibiting the expression of Wnt4, whereas its application and efficacy in treating human malignancies have not been reported (98).

Wnt5a and Wnt5b are highly homologous proteins with 82% amino acid sequence identity. The orthologs of Wnt5a are evolutionarily conserved, whereas those of Wnt5b are significantly divergent (99). Traditionally, Wnt5a is believed to be the non-canonical Wnt ligand and activates Ca^2+^-dependent effectors and other non-canonical pathways through small Rho-GTPases and c-Jun-NH2-kinase (100). However, its role in the progression of CRC is complicated and seems to be contradictory. Several studies proved that Wnt5a was silenced in most CRC cell lines and specimens due to frequent methylation in its promoter region (41, 42), and Wnt5a acts as a tumor suppressor in human CRC by interfering with the canonical β-catenin signaling but activating the non-canonical signaling pathways (43, 44). The expression of Wnt5a was negatively correlated with the degree of tumor differentiation and the aggressive behavior (41, 101). Meanwhile, promoter methylation of Wnt5a was strongly associated with the microsatellite instability status of patients with CRC, and multiple histone modifications of Wnt5a were involved in Wnt5a silencing and might promote colon cancer metastasis, providing evidence that epigenetic events may promote Wnt5a-mediated signaling in CRC (102, 103). On the contrary, other studies demonstrated that Wnt5a was upregulated consistently in intestinal polyps and tumor samples, and increased Wnt5a expression predicted the early recurrence or metastasis in colon cancer patients (45, 46). Wnt5a could also promote the migration of CRC cells by activating Fzd7-driven non-canonical Wnt signaling and enhance the cell stemness of CRC through activating the canonical Wnt signaling (47, 48). Furthermore, Jiang et al. demonstrated that a higher Wnt5a methylation status could predict a better drug response and longer progression-free survival in 5-fluorouracil-treated CRC patients (104). Until recently, Bauer et al. found two isoforms of Wnt5a protein with opposite functions in cancers (105), and a subsequent study proved that the simultaneous reactivation of the downregulated Wnt5a-long mRNA isoform and knockdown of the upregulated Wnt5a-short mRNA isoform could induce the apoptosis of CRC cells by silencing the expression of β-catenin, providing a reasonable explanation for the obscure role of Wnt5a in CRC previously (106). Wnt5b plays a pivotal role during embryonic gut development (51), and its expression level is increased significantly in ulcerative colitis and CRC samples (49, 50). Moreover, the Wnt5b rs2010851 polymorphism predicts a high risk of tumor recurrence in patients with advance-stage colon cancer (107). Overexpression of Wnt5b increased the proliferation, migration, and invasion of CRC cells through activating the non-canonical Wnt/JNK signaling (50). Meanwhile, Wnt5b exosome released from CRC cells could stimulate the migration and the proliferation of other cancer cells in a paracrine manner (108). In contrast, downregulating Wnt5b signaling pathway by the knockdown of fatty acid synthase could contribute to the decrease in invasion and metastasis of CRC cells, indicating that targeting Wnt5b is a promising approach to treat CRC.

Wnt6 is most homologous to Wnt1, with 43% amino acid sequence identity. It is apparently upregulated during intestinal development and regeneration as well as in CRC cells (51, 52). Overexpression or activation of Wnt6 could promote CRC development via activating the canonical Wnt signaling (53, 54). Moreover, a significant upregulation of methylation in *Wnt6* gene was also detected in CRC samples (109), and the Wnt6 rs6747776 polymorphism may participate in the increased risk of CRC associated with excessive saturated fat intake (110). These findings indicate that Wnt6 mainly functions as a carcinogenic factor in CRC progression and could be utilized as a potential therapeutic target.

Wnt7a and Wnt7b share 78% amino acid sequence identity. Wnt7a is considered to be a crucial ligand for the canonical Wnt signaling, whereas its role in tumorigenesis is also controversial. Wnt7a promotes the progression of bladder, ovarian, tongue, and pancreatic cancers. However, it inhibits the growth of lung, breast, endometrial, renal, and gastric cancers, and there are few studies about its effect on CRC development. A significant increase of Wnt7a expression was detected in ulcerative colitis specimens and CRC cells, implying a potential carcinogenic effect (49, 55), whereas Becer et al. found that *Colchicum pusillum* exerted anticancer activities through activating the Wnt7a/β-catenin pathway, and another study also proved that the loss of Wnt7a expression contributed to tumor progression and predicted a poor prognosis of CRC, which indicates a protective role of Wnt7a during CRC carcinogenesis (56, 111). Thus, the exact role of Wnt7a in CRC progression still needs to be studied further. Wnt7b is weakly expressed in adult lung, brain, and prostate. Several studies have documented that the upregulation of Wnt7b was necessary for the growth, invasion, and metastasis of breast cancer and pancreatic adenocarcinoma through activating the canonical Wnt signaling (112, 113). Additionally, Wnt7b could promote the growth of prostate cancer through activating the non-canonical pathways (114). However, there has not been any report about the role of Wnt7b in the tumorigenesis of CRC, except a recent study which claimed that Wnt7b was highly expressed in CRC tissues by using bioinformatics analysis.

Wnt8a and Wnt8b are also secreted proteins with 63% amino acid sequence identity (115). At present, there is no report about the role of Wnt8a in cancer; only one study indirectly demonstrated that clofibrate could abrogate the binding of nuclear factor-κB to the Wnt8a promoter and downregulate the expression of Wnt8a and Wnt/β-catenin signaling activity, which ultimately sensitized pancreatic cancer cells to radiation (116). Compared with Wnt8a, Wnt8b attracts a little more attention due to its more conserved orthologs. However, more researches are focused on its indispensable role in the formation of certain organs (117, 118), and only one study showed that Wnt8b was significantly upregulated in gastric cancer cell lines and most primary gastric cancer tissues (119). Therefore, whether the upregulation of Wnt8a and Wnt8b also promote the progression of CRC remains an intriguing question.

Wnt9a and Wnt9b share 63% amino acid sequence identity. Wnt9a, known as Wnt14 formerly, is required for chondrogenesis and aortic amplification and identified as the ligand for both canonical and non-canonical Wnt signaling pathways (120, 121). Wnt9a is considered to be a tumor suppressor gene during CRC development. Ali *et al*. found that the LiCl-mediated induction of Wnt9a could suppress CRC proliferation and promote apoptosis through inhibiting the expression and the active form of β-catenin (58). Furthermore, hypermethylation and the resultant low expression of Wnt9a occur frequently in primary colon cancer and corresponding cell lines (57), suggesting that activating the Wnt9a-mediated pathway may have a therapeutic effect on colorectal cancer. Wnt9b was known as Wnt14b previously, and the Wnt9b-mediated activation of canonical and non-canonical Wnt pathways is required for the organogenesis of the mammalian urogenital system and nasal and maxillary processes (122–124). However, little is known about the role of Wnt9b in tumorigenesis, and only one study indirectly reported that the expression of Wnt9b was downregulated by a cancer-preventing glycoconjugate in CRC cells (59), indicating a potential carcinogenic property of Wnt9b.

Wnt10a and Wnt10b are closely related Wnts with 62% amino acid sequence identity. It is believed that they are notably expressed in various tissues for their formation through a β-catenin-dependent pathway (125, 126). Several studies demonstrated that Wnt10a was highly expressed in CRC tissues and several corresponding cell lines, and a higher Wnt10a expression level was associated with an advanced tumor stage. Hence, it is not surprising that the knockdown of Wnt10a could suppress the proliferation and the invasiveness of CRC cells through inactivating the canonical Wnt signaling (60, 61). However, a recent result was counter to previous findings which demonstrated a reduced expression of Wnt10a and a negative correlation between its expression and methylation in CRC tissues. Moreover, a higher Wnt10a methylation level was detected in CRC patients with advanced age, with distant metastasis, and diagnosed with mucinous adenocarcinoma (62). An explanation for this contradictory observation could be the different types of tissues collected in different groups. Additionally, polymorphisms of Wnt10a gene were strongly associated with the upper tertile of saturated fat intake and the resulting increase in CRC adenoma risk (110). Wnt10b also takes part in the progression of several digestive system malignancies, such as gastric, liver, and colon cancers (127–129). In human CRC cells, overexpression of the antineoplastic miR-148a could suppress cellular invasion and migration as well as tumor growth *in vivo via* blocking Wnt10b expression and β-catenin signaling activities (63). However, the precise role of Wnt10b in oncogenesis is not completely consistent. In the study of Yoshikawa et al., upregulated Wnt10b was found to activate the β-catenin/TCF pathway. Unexpectedly, it also suppressed the growth rate of HCC cells and tumorigenicity in nude mice through a β-catenin-independent mechanism, and the authors finally found that the fibroblast growth factor family proteins were the crucial factors to switch Wnt10b from its growth-suppressive effects to growth-stimulatory ones (127). These observations suggest that the role of Wnt10b remains obscure and still needs to be elucidated by further studies.

Wnt11 is most homologous to Wnt4, with 41% amino acid sequence identity. Initially, it was identified as a non-canonical Wnt ligand, and its characteristics and function have been summarized elaborately by Onganer et al. (130). The role of Wnt11 in CRC was firstly documented due to its high expression levels in some colorectal adenocarcinomas (64). Then, Wnt11 was found to stimulate the proliferation and the transformation abilities of intestinal epithelial cells by activating the non-canonical Wnt signaling pathway (65). Consistently, the expression of Wnt11 was obviously upregulated in patients with recurrence than those without, and Wnt11-transfected CRC cells showed increased phenotypes of tumors (131). Furthermore, Wnt11 was identified as a target of estrogen-related receptor α/β-catenin complex and increased the migratory capacity of CRC cells in an autocrine manner (132). On the contrary, Wnt11 was also involved in the maintenance of intestinal homeostasis by protecting intestinal epithelial cells from the invasion of pathogenic bacteria and suppressing the inflammation and the consequent apoptosis (133). In general, these findings indicate that Wnt11 may act as a tumor promoter in CRC progression and can be used as a cancer drug target.

Wnt16 shows no homology to any other Wnts but generates two mRNA isoforms, Wnt16a and Wnt16b. They are only different in the sequences of 5′-untranslational region and the first exons. Wnt16a is only expressed highly in pancreas, whereas Wnt16b is widely distributed in many organs such as kidney, brain, and heart (134). Therefore, most reports about Wnt16 mainly refer to Wnt16b. It is now accepted that Wnt16 contributes to skeletal development and postnatal bone homeostasis *via* activating the canonical and the non-canonical Wnt signaling cascades (135). Wnt16 is overexpressed in gastric adenocarcinoma, leiomyoma, and head and neck squamous cell carcinoma tissues (136, 137). Upregulation of Wnt16 induced by estrogen and progesterone treatment in uterine leiomyoma stem cells could promote the growth of uterine leiomyomas through activating the canonical Wnt pathway in a paracrine manner (138), and the enhanced β-catenin activities initiated by Wnt16 in prostate cancer cells could promote the malignant phenotypes and chemoresistance through preventing cell death (139). On the contrary, silencing Wnt16 by miR-374b could suppress the cellular proliferation and promote the chemotherapeutic agent-induced apoptosis in T-cell lymphoblastic lymphoma (140). However, there is still no direct evidence about the role of Wnt16 in colorectal carcinogenesis, except that a study indirectly reported that an ellagic acid derivative performed an antitumor action in CRC cells by downregulating the expression of Wnt16 in a dose-dependent manner (66), indicating a potential carcinogenic role of Wnt16 during tumorigenesis. Therefore, in-depth studies are still required to elucidate its role in CRC progression.

## Opportunities and Challenges in Developing Wnt-based Therapeutics for CRC

According to the above information, most Wnts serve as primary determinants of colorectal carcinogenesis, and targeting the Wnt signaling pathway could be a promising therapeutic approach for CRC. For detailed information of targeting the Wnt signaling pathway in CRC, please refer to reviews by Sawa et al. and Bahrami et al. (141, 142). Herein we only give an overview of the current strategies to develop drugs directed at oncogenic Wnts for CRC treatment.

As mentioned earlier, porcupine is essential for the palmitoylation, secretion, and biological activity of Wnts. Theoretically, inhibiting its enzymatic activity could block all Wnt-driven cancers, and several small molecular inhibitors targeting porcupine have been developed for cancer treatment, including LGK974 (WNT974), Wnt-C59, ETC-159, IWP-O1, and GNF-6231 (143). Wnt-C59 and GNF-6231 are highly potent and orally available porcupine inhibitors capable of preventing the progression of mammary tumors in mice by downregulating Wnt1-mediated canonical signaling (144, 145). The pharmacological inhibition of the canonical Wnt signaling by Wnt-C59 and LGK974 could augment the cytotoxic effects of DNA-alkylating drug in CRC cells (146). It has been shown that Wnts secreted by fibroblast-exosomes protected differentiated CRC cells against chemotherapy, and their expression levels were correlated with the poor prognosis of patients with CRC. Therefore, blocking Wnt secretion by LGK974 treatment could diminish the clonogenic capacity and drug resistance of CRC cells *in vitro* and *in vivo* through decreasing the proportion of exosome Wnts (147). A phrase I trial of LGK974 is strikingly ongoing in patients with malignancies dependent on Wnts (NCT01351103), including BRAF mutant CRC. In addition, an orally available ETC-159 was proved to have a robust activity in CRC with RSPO mutations (148).

Furthermore, competitive receptors for Wnts were also explored to bind and sequester the free Wnts. Ipafricept (OMP-54F28) is a fusion protein that competes with native Fzd8 receptor for Wnts binding and blocks tumor growth in Wnt1-induced mice through antagonizing Wnt signaling. Moreover, ipafricept also impeded the growth of several solid tumors and selectively reduced the frequency of cancer stem cells (149). Intriguingly, in a phase I trial (NCT01608867) in patients with advanced solid tumors, ipafricept was well-tolerated with manageable toxicities, and a prolonged survival time was observed in a germ cell cancer and two desmoid tumor patients (150). Moreover, three phrase Ib clinical trials of ipafricept have just completed assessing its curative effect combined with different chemotherapeutic drugs in pancreatic cancer, ovarian cancer, and hepatocellular cancer. Additionally, some antibody-based inhibitors against specific Wnts have been produced to sequester the free Wnts. In the study of He et al., a monoclonal anti-Wnt1 antibody induced the apoptosis of human CRC cells expressing Wnt1 and showed great efficacy in treating primary tissue samples from patients with advanced CRC (79). Furthermore, the Wnt1 antibody also demonstrated an inhibitory effect on mesothelioma cells and the tumor growth of nude mice implanted with non-small cell lung cancer (NSCLC) cells. The inhibition of Wnt2 signaling by Wnt2 monoclonal antibody also similarly induced cell apoptosis and inhibited the tumor growth of several malignancies including melanoma, pleural mesothelioma, and NSCLC (151).

In another approach, some natural compounds targeting Wnt signaling have shown potential application values in cancer treatment, and several reviews have made elegant descriptions (149). Flavonoids deserve more attention due to the efficiently protective role against CRC through modulating the Wnt signaling pathways (152). For example, taxifolin was shown to induce cell cycle arrest and tumor regression in CRC cells by targeting the canonical Wnt signaling (153). By the same token, as another extensively studied flavonoid, (-)-epigallocatechin-3-gallate (EGCG) is abundantly distributed in green tea and exerts a preventive and therapeutic effect on CRC by promoting the degradation of intracellular β-catenin and the subsequent silence of Wnt/β-catenin-dependent genes (154). Interestingly, the effect of EGCG on CRC could be attributed to the inhibition on CRC stem cells by suppressing the canonical Wnt signaling (155). Furthermore, other natural compounds such as calycosin, isobavachalcone, resveratrol, etc., were also proved to suppress the malignant phenotypes of CRC via inhibiting the Wnt signaling pathways (156–158), providing more feasible therapeutic options for CRC treatment. However, few natural compounds have been proven to suppress the development of CRC by directly targeting Wnts, and none of them is currently undergoing clinical testing.

Despite the great advantages of the abovementioned approaches targeting Wnts, several potential pitfalls are still blocking their clinical application in cancer treatment. For instance, the ubiquitous Wnt signaling networks and numerous effects complicate the blockade of Wnt signaling. Different Wnts may exert opposite functions during CRC development, and inhibition of a specific Wnt alone is insufficient to curb CRC progression. Moreover, data on the pharmacokinetic parameters and efficacy of most reported natural compounds are far from sufficient. Additionally, a potential off-target effect of the manipulation of Wnt signaling is common for some therapeutic drugs. For example, inhibitors or antibodies targeting Wnt-dependent components in canonical Wnt signaling, such as Fzd receptors, may have no ability to block non-canonical Wnt signaling. Although porcupine inhibitors offer an approach to overcome this limitation, they have potential toxicity to the gastrointestinal tract and could lead to alteration in bone remodeling due to the essential role of Wnts for the maintenance of normal tissue homeostasis (150). More importantly, most of CRC attributed to the mutations of downstream components in Wnt signaling, such as APC and β-catenin, might be insensitive to porcupine inhibitors (159). Therefore, only therapies with the combination of drugs targeting Wnts, mutated downstream components in Wnt signaling, and conventional chemotherapeutics could result in a cooperative inhibition of CRC progression. Fortunately, all these obstacles are being overcome with great efforts of scientists and the in-depth understanding of the Wnt signaling pathways, and Wnt-based therapeutics are still promising and will definitely provide therapeutic benefits for patients with CRC and other cancers.

## Conclusion

The Wnt signaling pathway is essential for the regulation of embryogenesis and tissue homeostasis. However, dysregulation of the Wnt signaling pathway has been identified as the pathological basis of many human malignancies including CRC. Therefore, modulating this pathway is always a hotspot in the tumor field, and numerous approaches have been explored in preclinical and early clinical studies. In this review, we specifically described the recent findings on the structure and the maturation process of Wnts, discussed their functions during the tumorigenesis of human CRC separately, and summarized the current therapeutics targeting Wnts in CRC treatment and the existing challenges. We hope that the discussion of this topic will increase the knowledge of Wnts in CRC development and arouse the interest of researchers to design novel Wnt-based therapeutic strategies for CRC.

## Author Contributions

XN wrote the manuscript. XN and HL revised the manuscript. HL and LL edited the manuscript and drew the figures. W-DC and Y-DW edited, modified, and revised the manuscript. XN, W-DC, and Y-DW secured funding for this work. All authors contributed to the article and approved the submitted version.

## Conflict of Interest

The authors declare that the research was conducted in the absence of any commercial or financial relationships that could be construed as a potential conflict of interest.
